# Reporting of patient-centred outcomes in heart failure trials: are patient preferences being ignored?

**DOI:** 10.1007/s10741-015-9476-9

**Published:** 2015-02-18

**Authors:** Jeanet W. Blom, Maya El Azzi, Daisy M. Wopereis, Liam Glynn, Christiane Muth, Mieke L. van Driel

**Affiliations:** 1 Department of Public Health and Primary Care (V0-P), Leiden University Medical Center, Postbox 9600, 2300 RC Leiden, The Netherlands; 2 Discipline of General Practice, School of Medicine, The University of Queensland, Brisbane, Australia; 3 Discipline of General Practice, School of Medicine, National University of Ireland Galway, Galway, Ireland; 4 Institute of General Practice, Johann Wolfgang Goethe University, Frankfurt am Main, Germany

**Keywords:** Heart failure, Patient-reported outcomes, Patient-centred, Multimorbidity

## Abstract

Older people often suffer from multiple diseases. Therefore, universal cross-disease outcomes (e.g. functional status, quality of life, overall survival) are more relevant than disease-specific outcomes, and a range of potential outcomes are needed for medical decision-making. To assess how patient-relevant outcomes have penetrated randomized controlled trials (RCTs), reporting of these outcomes was reviewed in heart failure trials that included patients with multimorbidity. We systematically reviewed RCTs (Jan 2011–June 2012) and evaluated reported outcomes. Heart failure was chosen as condition of interest as this is common among older patients with multimorbidity. The main outcome was the proportion of RCTs reporting all-cause mortality, all-cause hospital admission, and outcomes in four domains of health, i.e. functional, signs and symptoms, psychological, and social domains. Of the 106 included RCTs, 50 (47 %) reported all-cause mortality and cardiovascular mortality and 29 (27 %) reported all-cause hospitalization and cardiovascular hospitalization. Of all trials, 68 (64 %) measured outcomes in the functional domain, 80 (75 %) in the domain of signs and symptoms, 65 (61 %) in the psychological domain, and 59 (56 %) in the social domain. Disease-specific instruments were more often used than non-disease-specific instruments. This review shows increasing attention for more patient-relevant outcomes; this is promising and indicates more awareness of the importance of a variety of outcomes desirable for patients. However, patients’ individual goal attainments were universally absent. For continued progress in patient-centred care, efforts are needed to develop these outcomes, study their merits and pitfalls, and intensify their use in research.

**Electronic supplementary material:**

The online version of this article (doi:10.1007/s10741-015-9476-9) contains supplementary material, which is available to authorized users.

## Introduction

Clinical decisions in the management of chronic diseases are usually guided by disease-specific targets, such as symptom control, prevention of impaired organ function, achievement of targeted laboratory parameters, or disease-specific survival. However, in older people who often suffer from multiple diseases, universal cross-disease outcomes, such as functional status, quality of life (QoL), or overall survival, are more relevant, as different diseases and treatments may interact and a range of potential outcomes (desired and undesired) have to be taken into account in medical decision-making [1–3]. Improving the disease-specific outcomes of one disease may not prevent deterioration of outcomes of another disease, which can be more harmful and stressful to the patient [4]. Therefore, universal outcomes become more important and relevant to patients with multiple conditions. However, the same does not seem to apply to the research community, which generally remains focused on uni-dimensional disease-specific and often surrogate outcomes [5, 6] that may have little impact in the everyday lives of patients.

Older people can develop their own individual preferences for what treatment of their chronic disease(s) should achieve. For instance, daily functioning can become more important than survival or maintaining independence more important than prevention of disease [7].This has been taken into consideration in patient-centred care. Patient-centred care is respectful of and responsive to individual patient preferences, needs, and values. These patient values are integrated in good clinical decisions [8, 9]. Therefore, in patient-centred care, clinicians need to identify their patients’ preferred or priority outcomes in various domains of health on an ongoing basis and adjust their therapy accordingly.

To facilitate evidence-based clinical decision-making in patient-centred care, ideally outcomes should therefore be goal-oriented, specifying patients’ own individual goals [7]. Currently, however, the use of measures such as goal attainment scales is mainly restricted to rehabilitation medicine [10, 11]. As long as individual goal attainment remains difficult to measure, research should provide clinicians with a range of disease-specific as well as non-disease-specific patient-relevant outcomes (i.e. outcomes that are meaningful to patients), and estimate or discuss the associations between them [12]. This will enable clinicians to focus on improving functional status when this is the patient’s preference, or on improving survival when this meets the patient’s priority. Evidence to support such decisions, especially in patients with multiple diseases, is sparse. We hypothesize that, although patient-relevant outcomes such as all-cause mortality are used in research, patient-relevant outcomes in other domains of health and wellbeing are underrepresented. Apart from outcomes on all-cause hospital admission and survival, patient-relevant outcomes in other domains of health are of interest. These can be classified into five dimensions: functional (activities of daily living), somatic (signs and symptoms), psychological, social, and communicative. This classification was developed in rehabilitation medicine and has been applied extensively in Dutch nursing home care [13].

In order to assess the range of reported outcomes and to study whether patient-relevant outcomes in a variety of health domains have been measured by randomized controlled trials (RCTs), we reviewed the reporting of patient-relevant (cross-disease) outcomes in RCTs that included patients with chronic heart failure. We chose this condition as a model, as patients with heart failure are generally older people who are more likely to suffer from multiple conditions [14].

## Methods

### Research question

To examine which patient-relevant outcomes are reported in RCTs in patients with chronic heart failure, RCTs published from 1 January 2011 to 1 June 2012 were reviewed and the reported outcomes were evaluated.

### Search strategy

The search for RCTs was performed by an expert librarian. PubMed was searched for RCTs on patients with heart failure using the following search strategy: (“heart failure”[Major] OR “heart failure”[ti] OR “Cardiac Failure”[ti] OR “Myocardial Failure”[ti] OR “Heart Decompensation”[ti]) AND (“Randomized Controlled Trial”[Publication Type] OR “Randomized Controlled Trial”[ti] OR “RCT”[ti] OR “Controlled Clinical Trial”[Publication Type] OR randomized[ti] OR randomised[ti] OR placebo[ti] OR randomly[ti] OR trial[ti]).

### Eligibility criteria

Studies were eligible if they reported a phase 3 or 4 RCT in adult patients with chronic or acute heart failure. Studies reporting only subgroup analysis of an RCT were excluded. No limitations on interventions, patient groups, or language were applied. RCTs were not excluded on the basis of methodological quality of the study.

### Screening and data extraction

RCTs were selected independently by two authors (JB, ME) by screening title and abstract and full article if necessary. Any discrepancies were resolved by consensus with a third author (MVD). Two researchers independently extracted information (BV, ME). In the case of disagreement on extracted data, consensus was reached by discussion with a third author (JB).

Variables collected were as follows: sample size, intervention and control group, mean age, proportion of male subjects, excluded and registered co-morbidity, and assessed outcomes (primary outcomes, as well as secondary outcomes). The data were extracted into pre-specified tables.

### Categorizing of outcomes used in the studies

In accordance with the aim to review a diversity of patient-relevant outcomes, we defined the outcomes of interest as follows: all-cause mortality, all-cause hospital admission, and disease-specific and non-specific outcomes representing the Bangma domains [13]. These domains represent 5 domains of health: functional (activities of daily living), somatic (signs and symptoms), psychological, social, and communicative. We combined the social and communicative domain into one domain. This classification has been developed in rehabilitation medicine and has been applied extensively in Dutch nursing home medicine. It is an aid to cover all health domains while making an inventory of existing problems relevant to the patient. The patient-relevant outcomes could be self-reported (e.g. QoL questionnaires or self-reported symptoms) or could be observed (e.g. the 6-m walking test, or NYHA class). Other reported outcomes concerning caregivers, costs, perception of care, self-care or care knowledge, and surrogate outcomes such as biomarkers or intermediate outcomes (e.g. ejection fraction measured by echocardiography) were not analysed. In addition to the above-mentioned outcomes of interest, we also checked whether goal-oriented outcomes were used. As goal-oriented outcomes, we considered outcomes that took into account patient’s preferences, such as the achievement of individually agreed health care goals (e.g. goal attainment scales).

The reported outcomes were scrutinized to examine their coverage of the Bangma domains [13] by two researchers (BdV, ME). Per instrument, each of the items/questions was allocated to a certain Bangma domain by two researchers independently. The social and communicative domain was aggregated as a social domain. The psychological domain concerned psychological and cognitive issues. Disagreement was resolved in discussion with a third author (JB).

The main outcome was the proportion of RCTs reporting all-cause mortality, all-cause hospital admission, and outcomes in the Bangma domains (functional, signs and symptoms, psychological, and social).

No distinction was made between primary and secondary outcomes as reported in the studies, as we did not aim to synthesize the data quantitatively.

### Data analysis

The number and percentage of studies using outcome measures in the above-mentioned dimensions are described.

As QoL measures were often used and cover all dimensions, we first tabulate QoL measures (disease-specific and non-specific) and subsequently other outcome measures covering at least one dimension (disease-specific and non-specific).

## Results

### Characteristics of the included studies

**Fig. 1:**
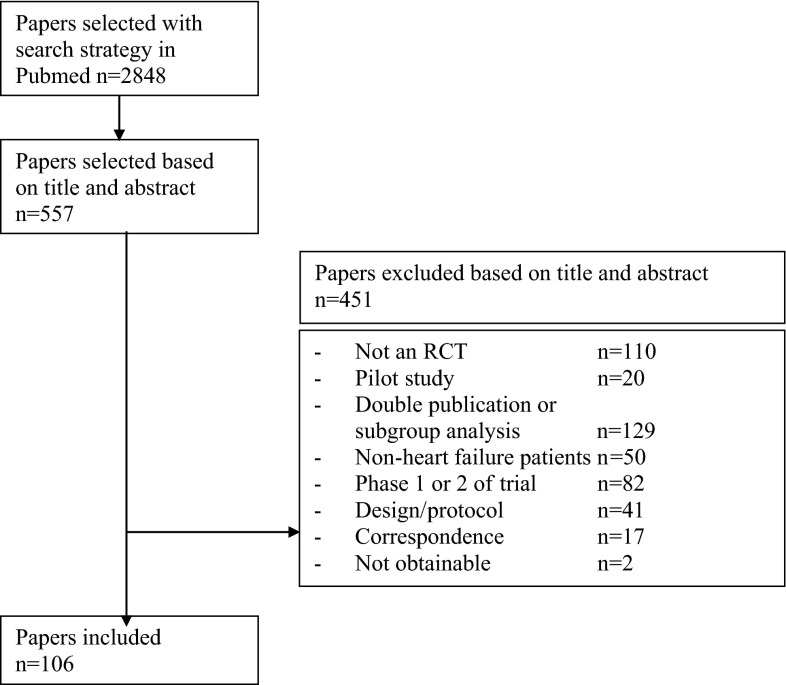
Inclusion of the studies

Figure 1 shows the selection of the 106 RCTs included in this review, and Table 1 summarizes the characteristics. Of all included trials, 77 (73 %) had a population of ≥50 patients, of which 60 trials had a population of ≥100 patients. Of all trials, 29 % concerned drug interventions and the remainder investigated non-drug interventions (e.g. exercise and diet) or health service interventions. Most trials (72 %) included patients from all NYHA classes.

**Table 1:** Characteristics of the trials (*n* = 106), reporting of comorbidity, and exclusion based on comorbidity

	No. of studies (%)
Mean age in years (range)^a^	67.6 (37.2–80.4)
Proportion of male subjects^a^	66.7
Chronic heart failure	98 (92)
*Type of intervention*	
Drug intervention	31 (29)
Non-drug intervention (e.g. surgery, exercise and dietary interventions)	48 (45)
Health service intervention (e.g. nurse-led (tele)monitoring, multidisciplinary monitoring, education on CHF management)	27 (26)
Sample size median (IQR)	111 (46–265)
*NYHA classification used in inclusion*	
Only I–II	3 (3)
Only II–III	22 (21)
Only III–IV	5 (5)
All	76 (72)
*Excluded conditions*	
Diabetes mellitus	12 (11)
Hypertension	10 (9)
Other cardiovascular disease including dyslipidaemia	71 (67)
Other non-cardiovascular disease	72 (68)
*Type of conditions reported*	
Diabetes mellitus	63 (59)
Hypertension	58 (55)
Other cardiovascular disease including dyslipidaemia	58 (55)
Other non-cardiovascular disease	42 (40)

^a^Calculated over all studies

### Reported outcomes in heart failure RCTs

A total of 50 (47 %) trials reported all-cause and cardiovascular mortality, 12 (11 %) reported only all-cause mortality, and 5 (5 %) only cardiovascular mortality. For hospitalization, this was 29 (27 %) for all-cause and cardiovascular, 3 (3 %) for only all-cause, and 26 (25 %) for only cardiovascular hospitalization.

A total of 68 (64 %) trials studied outcomes in the functional domain, 80 (75 %) in the domain of signs and symptoms, 65 (61 %) in the psychological domain, and 59 (56 %) in the social domain. For the group of drug trials this was 39, 74, 39, and 32 % respectively, for health service interventions this was 67, 67, 63, and 56 %, and for the remaining non-drug trials this was 79, 81, 75 and 71 %. No goal-oriented outcomes were used in any of the studies.

In three RCTs (3 %), only surrogate outcomes (e.g. cardiopulmonary exercise tests, blood pressure, pulse rate, ECGs, pro-BNP and other biomarkers) were reported.

### Outcome instruments used

In total, 60 (57 %) trials used QoL instruments; 9 studies used two QoL outcomes, and two studies used three QoL outcomes. Disease-specific QoL instruments were applied more than twice as often as non-disease-specific instruments: 48 (45 %) versus 22 (21 %) trials. Of the instruments other than QoL, in 51 (48 %) trials disease-specific instruments for outcome measurement were used and in 38 (36 %) trials non-disease-specific instruments were used.

Table 2 provides an overview of the instruments used in more than one study and covering all Bangma domains. QoL scales contain items on all Bangma domains.

**Table 2:**
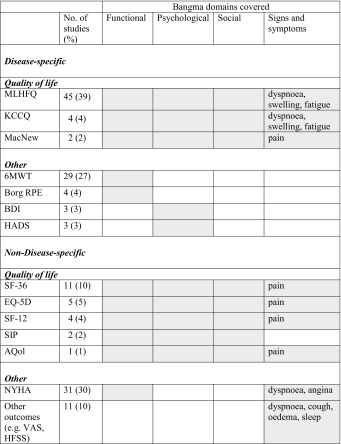
Instruments used in more than one study and covering the Bangma domains

*MLHFQ* minnesota living with heart failure questionnaire [30], *KCCQ* Kansas City cardiomyopathy questionnaire [31], *McNew* McNew QoL after myocardial infarction questionnaire [32], *SF* short-form 12 and 36 [33], *EQ5D* Euro quality-of-life 5D [34], *AQoL* assessment quality of life [35], *NYHA* New York Heart Association, *VAS* visual analogue scale, *HFSS* heart failure symptom scale [36], *6MWT* six-minute walking test [37], *RPE* rating of perceived exertion [38], *BDI* Beck’s depression inventory [39], *HADS* hospital anxiety and depression scale [40]

Other disease-specific and non-specific patient-relevant outcomes relating to one or more of the domains which were used in a single study only (and not included in Table 2) were as follows: various outcomes for depressive symptoms or cognition (*n* = 9 trials), various outcomes for activity or energy expenditure (*n* = 5 trials), outcomes for heart failure symptoms (*n* = 4 trials), outcomes related to sleep quality (*n* = 3 trials), outcomes measuring treatment satisfaction (*n* = 2 trials), and outcomes related to patients’ perception of control over their condition (*n* = 1 trial).

## Discussion

### Main findings

In this review of heart failure RCTs, we found a relatively broad range of potentially patient-relevant outcomes addressing mortality, hospitalization, and outcomes in the Bangma health domains. This finding is promising and may demonstrate an awareness of the importance of a variety of outcomes that are desirable for patients. However, none of the trials reported goal attainment in accordance with patients’ individual preferences. Whereas all-cause mortality and hospitalization were more frequently measured than their disease-specific counterparts, the majority of patient-reported outcomes measured were still based on disease-specific instruments. Almost two-thirds of the trials studied outcomes in at least one of the four domains of health (i.e. functional domain, domain of signs and symptoms, psychological domain, or social domain): of these, signs and symptoms were by far the most investigated, and functioning and the psychological and social domains were the least investigated. Remarkably, non-drug trials used other patient-relevant outcomes than signs and symptoms about twice as often than drug trials.

Although many of the trials applied QoL instruments that cover most or all domains to some extent, the aggregation of different domains in a sum score hampers a differentiated conclusion to inform medical decision-making. Nevertheless, it has been argued that QoL measures should be used more often in heart failure trials [15, 16] in order to incorporate outcomes that are relevant to patients, in addition to mortality and hospital admission. However, Gill et al. [17] argue that QoL measures do not include patients’ opinions and reactions and therefore do not aim at the correct target; this conclusion was confirmed by Dunderdale et al. [18]. Most of the QoL instruments are not patient-centred and restrict the patient’s choice by imposing standard models of QoL and preselected domains on the individual. Furthermore, QoL instruments have mainly been developed and validated in younger populations and tend to be phrased mainly in relation to physical function, thus underestimating QoL in older persons whose physical function is likely to be not as good as that of younger people [19]. QoL of older people (e.g. most patients with chronic heart failure) is considered a multidimensional construct that includes objective indicators and subjective evaluations related to developmental processes of growth, maintenance, and resilience, as well as management of loss, which have not been adopted by the QoL instruments used in the RCTs included in this review [20].

In the reviewed trials, important specific outcomes (e.g. dyspnoea, oedema and fatigue) were mainly evaluated by the QoL questionnaires used; however, this method of evaluating heart failure outcomes is reported to be inadequate [21]. In addition, pain is generally not included in heart failure-specific QoL measures, as it is not a symptom caused by heart failure. Nevertheless, pain is very common in heart failure patients due to the high prevalence of (painful) comorbidities [22]. Another disadvantage of (in particular) disease-specific QoL instruments is that the questions relate to the disease under study, in this case heart failure. For example, a question about depressive feelings links these feelings to heart failure: ‘*Did your heart failure prevent you from living as you wanted during the past month (4* *weeks) by making you feel depressed?*’ In this way, general feelings of depression unrelated to heart failure might be missed [23].

To the best of our knowledge, this is the first study to classify outcomes into patient-relevant domains of health. We used the Bangma criteria that provide a holistic framework that has been used and validated in rehabilitation medicine. The Bangma model [13] is designed to support problem-based care as opposed to disease-oriented care and lists all clinically relevant problem areas of the patient: activities of daily living, signs and symptoms, psychological, social, and communicative domains. This model is similar to the composite measure recommended by the National Institute on Aging to monitor the health of older people with multiple chronic conditions [5].

### Strengths and limitations

We used Pubmed to select a systematic sample of RCTs over a certain period of time that included patients with heart failure and multiple diseases. Some RCTs might have been missed by not searching other databases such as Embase or Web of Science. However, our aim was not to conduct an exhaustive overview of RCTs including heart failure patients, but rather to capture a large sample of such studies. A strength of our study is that all selection and data extraction was conducted by two reviewers independently, which reduces the risk of bias.

In this review, although the attention paid to more patient-relevant outcomes is promising, this finding may be influenced by the choice of the primary condition. We chose heart failure as it is a common condition in older patients with multimorbidity. The association between heart failure and multimorbidity was reported more than a decade ago [14], and therefore, recent heart failure guidelines address multimorbidity more often than the guidelines for other diseases [24]. For these reasons, our results may be overly optimistic when applied to other chronic diseases where the debate about multimorbidity is still relatively young and may not have influenced the choice of outcomes in research.

### Conclusion and implications

Although an encouragingly high proportion of heart failure trials report patient-relevant outcomes, patients’ individual goal attainments were universally absent from all the trials included in this review. In practice, clinicians negotiate clinical management with their patients usually taking their individual preferences into account. However, in research we are still far from giving individual goals a priority. Some research groups have developed patient-reported outcomes that include patients’ goals [25–28]. However, their feasibility and completeness, especially for research purposes, is still suboptimal [29]. To make progress in patient-centred care, more studies are needed to further develop these outcomes, examine their merits and pitfalls, and intensify their use in research. Patients need to be centrally involved in the design, development, and testing of such goal-orientated outcome research methods.

## Electronic supplementary material

Below is the link to the electronic supplementary material.

Supplementary material 1 (DOCX 29 kb)
