# Supplementary material for: Reporting of patient-centred outcomes in heart failure trials: are patient preferences being ignored?
Source: Heart Fail Rev. 2015 Feb 18;20(4):385–92. doi: 10.1007/s10741-015-9476-9 (PMC4464642; doi:10.1007/s10741-015-9476-9)
Supplement: Supplementary file 1 — Supplementary material 1 (DOCX 29 kb) [file 10741_2015_9476_MOESM1_ESM.docx]

**Appendix**

Included studies

1. Abraham WT, Adamson PB, Bourge RC, Aaron MF, Costanzo MR, Stevenson LW, et al. Wireless pulmonary artery haemodynamic monitoring in chronic heart failure: a randomised controlled trial. Lancet. 2011 Feb 19;377(9766):658-66.

2. Acker MA, Jessup M, Bolling SF, Oh J, Starling RC, Mann DL, et al. Mitral valve repair in heart failure: five-year follow-up from the mitral valve replacement stratum of the Acorn randomized trial. The Journal of thoracic and cardiovascular surgery. 2011 Sep;142(3):569-74.

3. Babu AS, Maiya AG, George MM, Padmakumar R, Guddattu V. Effects of Combined Early In-Patient Cardiac Rehabilitation and Structured Home-based Program on Function among Patients with Congestive Heart Failure: A Randomized Controlled Trial. Heart views : the official journal of the Gulf Heart Association. 2011 Jul;12(3):99-103.

4. Baker DW, Dewalt DA, Schillinger D, Hawk V, Ruo B, Bibbins-Domingo K, et al. The effect of progressive, reinforcing telephone education and counseling versus brief educational intervention on knowledge, self-care behaviors and heart failure symptoms. Journal of cardiac failure. 2011 Oct;17(10):789-96.

5. Beyranvand MR, Khalafi MK, Roshan VD, Choobineh S, Parsa SA, Piranfar MA. Effect of taurine supplementation on exercise capacity of patients with heart failure. Journal of cardiology. 2011 May;57(3):333-7.

6. Bosnak-Guclu M, Arikan H, Savci S, Inal-Ince D, Tulumen E, Aytemir K, et al. Effects of inspiratory muscle training in patients with heart failure. Respiratory medicine. 2011 Nov;105(11):1671-81.

7. Chien CL, Lee CM, Wu YW, Wu YT. Home-based exercise improves the quality of life and physical function but not the psychological status of people with chronic heart failure: a randomised trial. Journal of physiotherapy. 2011;57(3):157-63.

8. Cleland JG, Calvert M, Freemantle N, Arrow Y, Ball SG, Bonser RS, et al. The Heart Failure Revascularisation Trial (HEART). European journal of heart failure. 2011 Feb;13(2):227-33.

9. Cowie A, Thow MK, Granat MH, Mitchell SL. A comparison of home and hospital-based exercise training in heart failure: immediate and long-term effects upon physical activity level. European journal of cardiovascular prevention and rehabilitation : official journal of the European Society of Cardiology, Working Groups on Epidemiology & Prevention and Cardiac Rehabilitation and Exercise Physiology. 2011 Apr;18(2):158-66.

10. Deswal A, Richardson P, Bozkurt B, Mann DL. Results of the Randomized Aldosterone Antagonism in Heart Failure with Preserved Ejection Fraction trial (RAAM-PEF). Journal of cardiac failure. 2011 Aug;17(8):634-42.

11. Domingo M, Lupon J, Gonzalez B, Crespo E, Lopez R, Ramos A, et al. [Noninvasive remote telemonitoring for ambulatory patients with heart failure: effect on number of hospitalizations, days in hospital, and quality of life. CARME (CAtalan Remote Management Evaluation) study]. Revista espanola de cardiologia. 2011 Apr;64(4):277-85.

12. Domingues FB, Clausell N, Aliti GB, Dominguez DR, Rabelo ER. Education and telephone monitoring by nurses of patients with heart failure: randomized clinical trial. Arquivos brasileiros de cardiologia. 2011 Mar;96(3):233-9.

13. Dungen HD, Apostolovic S, Inkrot S, Tahirovic E, Topper A, Mehrhof F, et al. Titration to target dose of bisoprolol vs. carvedilol in elderly patients with heart failure: the CIBIS-ELD trial. European journal of heart failure. 2011 Jun;13(6):670-80.

14. Felker GM, Lee KL, Bull DA, Redfield MM, Stevenson LW, Goldsmith SR, et al. Diuretic strategies in patients with acute decompensated heart failure. The New England journal of medicine. 2011 Mar 3;364(9):797-805.

15. Foley PW, Patel K, Irwin N, Sanderson JE, Frenneaux MP, Smith RE, et al. Cardiac resynchronisation therapy in patients with heart failure and a normal QRS duration: the RESPOND study. Heart (British Cardiac Society). 2011 Jul;97(13):1041-7.

16. Fragasso G, Salerno A, Lattuada G, Cuko A, Calori G, Scollo A, et al. Effect of partial inhibition of fatty acid oxidation by trimetazidine on whole body energy metabolism in patients with chronic heart failure. Heart (British Cardiac Society). 2011 Sep;97(18):1495-500.

17. Fumagalli S, Fattirolli F, Guarducci L, Cellai T, Baldasseroni S, Tarantini F, et al. Coenzyme Q10 terclatrate and creatine in chronic heart failure: a randomized, placebo-controlled, double-blind study. Clinical cardiology. 2011 Apr;34(4):211-7.

18. Gary RA, Cress ME, Higgins MK, Smith AL, Dunbar SB. Combined aerobic and resistance exercise program improves task performance in patients with heart failure. Archives of physical medicine and rehabilitation. 2011 Sep;92(9):1371-81.

19. Giglioli C, Landi D, Cecchi E, Chiostri M, Gensini GF, Valente S, et al. Effects of ULTRAfiltration vs. DIureticS on clinical, biohumoral and haemodynamic variables in patients with deCOmpensated heart failure: the ULTRADISCO study. European journal of heart failure. 2011 Mar;13(3):337-46.

20. Giraldi F, Cattadori G, Roberto M, Carbucicchio C, Pepi M, Ballerini G, et al. Long-term effectiveness of cardiac resynchronization therapy in heart failure patients with unfavorable cardiac veins anatomy comparison of surgical versus hemodynamic procedure. Journal of the American College of Cardiology. 2011 Jul 26;58(5):483-90.

21. Group TI. Effects of prolonged-release torasemide versus furosemide on myocardial fibrosis in hypertensive patients with chronic heart failure: a randomized, blinded-end point, active-controlled study. Clinical therapeutics. 2011 Sep;33(9):1204-13.

22. Guazzi M, Vicenzi M, Arena R, Guazzi MD. Pulmonary hypertension in heart failure with preserved ejection fraction: a target of phosphodiesterase-5 inhibition in a 1-year study. Circulation. 2011 Jul 12;124(2):164-74.

23. Guazzi M, Vicenzi M, Arena R, Guazzi MD. PDE5 inhibition with sildenafil improves left ventricular diastolic function, cardiac geometry, and clinical status in patients with stable systolic heart failure: results of a 1-year, prospective, randomized, placebo-controlled study. Circulation Heart failure. 2011 Jan;4(1):8-17.

24. Hartog JW, Willemsen S, van Veldhuisen DJ, Posma JL, van Wijk LM, Hummel YM, et al. Effects of alagebrium, an advanced glycation endproduct breaker, on exercise tolerance and cardiac function in patients with chronic heart failure. European journal of heart failure. 2011 Aug;13(8):899-908.

25. Januzzi JL, Jr., Rehman SU, Mohammed AA, Bhardwaj A, Barajas L, Barajas J, et al. Use of amino-terminal pro-B-type natriuretic peptide to guide outpatient therapy of patients with chronic left ventricular systolic dysfunction. Journal of the American College of Cardiology. 2011 Oct 25;58(18):1881-9.

26. Jensen CJ, Liadski A, Bell M, Naber CK, Bruder O, Sabin GV, et al. Echocardiography versus intracardiac electrocardiography-based optimization for cardiac resynchronization therapy : a comparative clinical long-term trial. Herz. 2011 Oct;36(7):592-9.

27. Karavidas A, Kapsimalis F, Lazaros G, Markozanes E, Arapi S, Cholidou K, et al. The impact of positive airway pressure on cardiac status and clinical outcomes in patients with advanced heart failure and sleep-disordered breathing: a preliminary report. Sleep & breathing = Schlaf & Atmung. 2011 Dec;15(4):701-9.

28. Karlstrom P, Alehagen U, Boman K, Dahlstrom U, group UP-s. Brain natriuretic peptide-guided treatment does not improve morbidity and mortality in extensively treated patients with chronic heart failure: responders to treatment have a significantly better outcome. European journal of heart failure. 2011 Oct;13(10):1096-103.

29. Koehler F, Winkler S, Schieber M, Sechtem U, Stangl K, Bohm M, et al. Impact of remote telemedical management on mortality and hospitalizations in ambulatory patients with chronic heart failure: the telemedical interventional monitoring in heart failure study. Circulation. 2011 May 3;123(17):1873-80.

30. Konstam V, Gregory D, Chen J, Weintraub A, Patel A, Levine D, et al. Health-related quality of life in a multicenter randomized controlled comparison of telephonic disease management and automated home monitoring in patients recently hospitalized with heart failure: SPAN-CHF II trial. Journal of cardiac failure. 2011 Feb;17(2):151-7.

31. Lainscak M, Podbregar M, Kovacic D, Rozman J, von Haehling S. Differences between bisoprolol and carvedilol in patients with chronic heart failure and chronic obstructive pulmonary disease: a randomized trial. Respiratory medicine. 2011 Oct;105 Suppl 1:S44-9.

32. Laoutaris ID, Dritsas A, Adamopoulos S, Manginas A, Gouziouta A, Kallistratos MS, et al. Benefits of physical training on exercise capacity, inspiratory muscle function, and quality of life in patients with ventricular assist devices long-term postimplantation. European journal of cardiovascular prevention and rehabilitation 2011 Feb;18(1):33-40.

33. Leventhal ME, Denhaerynck K, Brunner-La Rocca HP, Burnand B, Conca-Zeller A, Bernasconi AT, et al. Swiss Interdisciplinary Management Programme for Heart Failure (SWIM-HF): a randomised controlled trial study of an outpatient inter-professional management programme for heart failure patients in Switzerland. Swiss medical weekly. 2011;141:w13171.

34. MacDonald MR, Connelly DT, Hawkins NM, Steedman T, Payne J, Shaw M, et al. Radiofrequency ablation for persistent atrial fibrillation in patients with advanced heart failure and severe left ventricular systolic dysfunction: a randomised controlled trial. Heart (British Cardiac Society). 2011 May;97(9):740-7.

35. Maejima Y, Nobori K, Ono Y, Adachi S, Suzuki J, Hirao K, et al. Synergistic effect of combined HMG-CoA reductase inhibitor and angiotensin-II receptor blocker therapy in patients with chronic heart failure: the HF-COSTAR trial. Circulation journal 2011;75(3):589-95.

36. Marazzi G, Iellamo F, Volterrani M, Caminiti G, Madonna M, Arisi G, et al. Comparison of effectiveness of carvedilol versus bisoprolol for prevention of postdischarge atrial fibrillation after coronary artery bypass grafting in patients with heart failure. The American journal of cardiology. 2011 Jan 15;107(2):215-9.

37. Marazzi G, Volterrani M, Caminiti G, Iaia L, Massaro R, Vitale C, et al. Comparative long term effects of nebivolol and carvedilol in hypertensive heart failure patients. Journal of cardiac failure. 2011 Sep;17(9):703-9.

38. Marchenko A, Chernyavsky A, Efendiev V, Volokitina T, Karaskov A. Results of coronary artery bypass grafting alone and combined with surgical ventricular reconstruction for ischemic heart failure. Interactive cardiovascular and thoracic surgery. 2011 Jul;13(1):46-51.

39. Nodari S, Triggiani M, Campia U, Manerba A, Milesi G, Cesana BM, et al. Effects of n-3 polyunsaturated fatty acids on left ventricular function and functional capacity in patients with dilated cardiomyopathy. Journal of the American College of Cardiology. 2011 Feb 15;57(7):870-9.

40. O'Connor CM, Starling RC, Hernandez AF, Armstrong PW, Dickstein K, Hasselblad V, et al. Effect of nesiritide in patients with acute decompensated heart failure. The New England journal of medicine. 2011 Jul 7;365(1):32-43.

41. Otsu H, Moriyama M. Effectiveness of an educational self-management program for outpatients with chronic heart failure. Japan journal of nursing science : JJNS. 2011 Dec;8(2):140-52.

42. Oxberry SG, Torgerson DJ, Bland JM, Clark AL, Cleland JG, Johnson MJ. Short-term opioids for breathlessness in stable chronic heart failure: a randomized controlled trial. European journal of heart failure. 2011 Sep;13(9):1006-12.

43. Ozova EM, Kiiakbaev GK, Kobalava Zh D, Moiseev VS. [Effect of carvedilol and metoprolol R administered with or without atorvastatin on elastic properties of vascular wall and parameters of inflammation in patients with chronic heart failure of ischemic origin]. Kardiologiia. 2011;51(4):39-46.

44. Paterna S, Fasullo S, Parrinello G, Cannizzaro S, Basile I, Vitrano G, et al. Short-term effects of hypertonic saline solution in acute heart failure and long-term effects of a moderate sodium restriction in patients with compensated heart failure with New York Heart Association class III (Class C) (SMAC-HF Study). The American journal of the medical sciences. 2011 Jul;342(1):27-37.

45. Pihl E, Cider A, Stromberg A, Fridlund B, Martensson J. Exercise in elderly patients with chronic heart failure in primary care: effects on physical capacity and health-related quality of life. European journal of cardiovascular nursing : journal of the Working Group on Cardiovascular Nursing of the European Society of Cardiology. 2011 Sep;10(3):150-8.

46. Pouleur AC, Knappe D, Shah AM, Uno H, Bourgoun M, Foster E, et al. Relationship between improvement in left ventricular dyssynchrony and contractile function and clinical outcome with cardiac resynchronization therapy: the MADIT-CRT trial. European heart journal. 2011 Jul;32(14):1720-9.

47. Pressler SJ, Therrien B, Riley PL, Chou CC, Ronis DL, Koelling TM, et al. Nurse-Enhanced Memory Intervention in Heart Failure: the MEMOIR study. Journal of cardiac failure. 2011 Oct;17(10):832-43.

48. Ronn F, Kesek M, Karp K, Henein M, Jensen SM. Right ventricular lead positioning does not influence the benefits of cardiac resynchronization therapy in patients with heart failure and atrial fibrillation. Europace : European pacing, arrhythmias, and cardiac electrophysiology : journal of the working groups on cardiac pacing, arrhythmias, and cardiac cellular electrophysiology of the European Society of Cardiology. 2011 Dec;13(12):1747-52.

49. Sela N, Baruch N, Assali A, Vaturi M, Battler A, Ben Gal T. [The influence of medical art therapy on quality of life and compliance of medical treatment of patients with advanced heart failure]. Harefuah. 2011 Feb;150(2):79-83, 209.

50. Shalaby A, Atwood CW, Selzer F, Suffoletto M, Gorcsan Iii J, Strollo P. Cardiac resynchronization therapy and obstructive sleep-related breathing disorder in patients with congestive heart failure. Pacing and clinical electrophysiology : PACE. 2011 May;34(5):593-603.

51. Sharma GV, Woods PA, Lindsey N, O'Connell C, Connolly L, Joseph J, et al. Noninvasive monitoring of left ventricular end-diastolic pressure reduces rehospitalization rates in patients hospitalized for heart failure: a randomized controlled trial. Journal of cardiac failure. 2011 Sep;17(9):718-25.

52. Sidorenko GI, Komissarova SM, Zolotukhina SF, Petrovskaia ME. [he use ethylmethylhydroxypyridine succinate in the treatment of patients with heart failure]. Kardiologiia. 2011;51(6):44-8.

53. Tamborero D, Vidal B, Tolosana JM, Sitges M, Berruezo A, Silva E, et al. Electrocardiographic versus echocardiographic optimization of the interventricular pacing delay in patients undergoing cardiac resynchronization therapy. Journal of cardiovascular electrophysiology. 2011 Oct;22(10):1129-34.

54. Teffaha D, Mourot L, Vernochet P, Ounissi F, Regnard J, Monpere C, et al. Relevance of water gymnastics in rehabilitation programs in patients with chronic heart failure or coronary artery disease with normal left ventricular function. Journal of cardiac failure. 2011 Aug;17(8):676-83.

55. Thibault B, Ducharme A, Harel F, White M, O'Meara E, Guertin MC, et al. Left ventricular versus simultaneous biventricular pacing in patients with heart failure and a QRS complex >/=120 milliseconds. Circulation. 2011 Dec 20;124(25):2874-81.

56. Udelson JE, Bilsker M, Hauptman PJ, Sequeira R, Thomas I, O'Brien T, et al. A multicenter, randomized, double-blind, placebo-controlled study of tolvaptan monotherapy compared to furosemide and the combination of tolvaptan and furosemide in patients with heart failure and systolic dysfunction. Journal of cardiac failure. 2011 Dec;17(12):973-81.

57. van Veldhuisen DJ, Braunschweig F, Conraads V, Ford I, Cowie MR, Jondeau G, et al. Intrathoracic impedance monitoring, audible patient alerts, and outcome in patients with heart failure. Circulation. 2011 Oct 18;124(16):1719-26.

58. Volterrani M, Cice G, Caminiti G, Vitale C, D'Isa S, Perrone Filardi P, et al. Effect of Carvedilol, Ivabradine or their combination on exercise capacity in patients with Heart Failure (the CARVIVA HF trial). International journal of cardiology. 2011 Sep 1;151(2):218-24.

59. Voors AA, Dittrich HC, Massie BM, DeLucca P, Mansoor GA, Metra M, et al. Effects of the adenosine A1 receptor antagonist rolofylline on renal function in patients with acute heart failure and renal dysfunction: results from PROTECT (Placebo-Controlled Randomized Study of the Selective Adenosine A1 Receptor Antagonist Rolofylline for Patients Hospitalized with Acute Decompensated Heart Failure and Volume Overload to Assess Treatment Effect on Congestion and Renal Function). Journal of the American College of Cardiology. 2011 May 10;57(19):1899-907.

60. Wade MJ, Desai AS, Spettell CM, Snyder AD, McGowan-Stackewicz V, Kummer PJ, et al. Telemonitoring with case management for seniors with heart failure. The American journal of managed care. 2011 Mar;17(3):e71-9.

61. Yang QY, Lu S, Sun HR. Clinical effect of Astragalus granule of different dosages on quality of life in patients with chronic heart failure. Chinese journal of integrative medicine. 2011 Feb;17(2):146-9.

62. Yeh GY, McCarthy EP, Wayne PM, Stevenson LW, Wood MJ, Forman D, et al. Tai chi exercise in patients with chronic heart failure: a randomized clinical trial. Archives of internal medicine. 2011 Apr 25;171(8):750-7.

63. Zannad F, McMurray JJ, Krum H, van Veldhuisen DJ, Swedberg K, Shi H, et al. Eplerenone in patients with systolic heart failure and mild symptoms. The New England journal of medicine. 2011 Jan 6;364(1):11-21.

64. Zou X, Pan GM, Sheng XG. [Double blinded randomized and controlled study on treatment of chronic heart failure by nuanxin capsule]. Zhongguo Zhong xi yi jie he za zhi Zhongguo Zhongxiyi jiehe zazhi = Chinese journal of integrated traditional and Western medicine / Zhongguo Zhong xi yi jie he xue hui, Zhongguo Zhong yi yan jiu yuan zhu ban. 2011 Jan;31(1):19-22.

65. Agren S, Evangelista LS, Hjelm C, Stromberg A. Dyads affected by chronic heart failure: a randomized study evaluating effects of education and psychosocial support to patients with heart failure and their partners. Journal of cardiac failure. 2012 May;18(5):359-66.

66. Alves AJ, Ribeiro F, Goldhammer E, Rivlin Y, Rosenschein U, Viana JL, et al. Exercise training improves diastolic function in heart failure patients. Medicine and science in sports and exercise. 2012 May;44(5):776-85.

67. Angermann CE, Stork S, Gelbrich G, Faller H, Jahns R, Frantz S, et al. Mode of action and effects of standardized collaborative disease management on mortality and morbidity in patients with systolic heart failure: the Interdisciplinary Network for Heart Failure (INH) study. Circulation Heart failure. 2012 Jan;5(1):25-35.

68. Barker A, Barlis P, Berlowitz D, Page K, Jackson B, Lim WK. Pharmacist directed home medication reviews in patients with chronic heart failure: a randomised clinical trial. International journal of cardiology. 2012 Aug 23;159(2):139-43.

69. Belardinelli R, Georgiou D, Cianci G, Purcaro A. 10-year exercise training in chronic heart failure: a randomized controlled trial. Journal of the American College of Cardiology. 2012 Oct 16;60(16):1521-8.

70. Blumenthal JA, Babyak MA, O'Connor C, Keteyian S, Landzberg J, Howlett J, et al. Effects of exercise training on depressive symptoms in patients with chronic heart failure: the HF-ACTION randomized trial. JAMA : the journal of the American Medical Association. 2012 Aug 1;308(5):465-74.

71. Boyne JJ, Vrijhoef HJ, Crijns HJ, De Weerd G, Kragten J, Gorgels AP, et al. Tailored telemonitoring in patients with heart failure: results of a multicentre randomized controlled trial. European journal of heart failure. 2012 Jul;14(7):791-801.

72. Costanzo MR, Ivanhoe RJ, Kao A, Anand IS, Bank A, Boehmer J, et al. Prospective evaluation of elastic restraint to lessen the effects of heart failure (PEERLESS-HF) trial. Journal of cardiac failure. 2012 Jun;18(6):446-58.

73. Dekker RL, Moser DK, Peden AR, Lennie TA. Cognitive therapy improves three-month outcomes in hospitalized patients with heart failure. Journal of cardiac failure. 2012 Jan;18(1):10-20.

74. Del Sindaco D, Pulignano G, Di Lenarda A, Tarantini L, Cioffi G, Tolone S, et al. Role of a multidisciplinary program in improving outcomes in cognitively impaired heart failure older patients. Monaldi archives for chest disease. 2012 Mar;78(1):20-8.

75. Dendale P, De Keulenaer G, Troisfontaines P, Weytjens C, Mullens W, Elegeert I, et al. Effect of a telemonitoring-facilitated collaboration between general practitioner and heart failure clinic on mortality and rehospitalization rates in severe heart failure: the TEMA-HF 1 (TElemonitoring in the MAnagement of Heart Failure) study. European journal of heart failure. 2012 Mar;14(3):333-40.

1. DeWalt DA, Schillinger D, Ruo B, Bibbins-Domingo K, Baker DW, Holmes GM, et al. Multisite randomized trial of a single-session versus multisession literacy-sensitive self-care intervention for patients with heart failure. Circulation. 2012 Jun 12;125(23):2854-62.

77. Donner Alves F, Correa Souza G, Brunetto S, Schweigert Perry ID, Biolo A. Nutritional orientation, knowledge and quality of diet in heart failure: randomized clinical trial. Nutricion hospitalaria : organo oficial de la Sociedad Espanola de Nutricion Parenteral y Enteral. 2012 Mar-Apr;27(2):441-8.

78. Freyssin C, Verkindt C, Prieur F, Benaich P, Maunier S, Blanc P. Cardiac rehabilitation in chronic heart failure: effect of an 8-week, high-intensity interval training versus continuous training. Archives of physical medicine and rehabilitation. 2012 Aug;93(8):1359-64.

79. Gamez-Lopez AL, Bonilla-Palomas JL, Anguita-Sanchez M, Castillo-Dominguez JC, Arizon del Prado JM, Suarez de Lezo J. [Effects of three different disease management programs on outcomes in patients hospitalized with heart failure: a randomized trial]. Medicina clinica. 2012 Mar 3;138(5):192-8.

80. Healey JS, Hohnloser SH, Exner DV, Birnie DH, Parkash R, Connolly SJ, et al. Cardiac resynchronization therapy in patients with permanent atrial fibrillation: results from the Resynchronization for Ambulatory Heart Failure Trial (RAFT). Circulation Heart failure. 2012 Sep 1;5(5):566-70.

81. Homma S, Thompson JL, Pullicino PM, Levin B, Freudenberger RS, Teerlink JR, et al. Warfarin and aspirin in patients with heart failure and sinus rhythm. The New England journal of medicine. 2012 May 17;366(20):1859-69.

82. Khan FZ, Virdee MS, Palmer CR, Pugh PJ, O'Halloran D, Elsik M, et al. Targeted left ventricular lead placement to guide cardiac resynchronization therapy: the TARGET study: a randomized, controlled trial. Journal of the American College of Cardiology. 2012 Apr 24;59(17):1509-18.

83. Kommuri NV, Johnson ML, Koelling TM. Relationship between improvements in heart failure patient disease specific knowledge and clinical events as part of a randomized controlled trial. Patient education and counseling. 2012 Feb;86(2):233-8.

84. Landolina M, Perego GB, Lunati M, Curnis A, Guenzati G, Vicentini A, et al. Remote monitoring reduces healthcare use and improves quality of care in heart failure patients with implantable defibrillators: the evolution of management strategies of heart failure patients with implantable defibrillators (EVOLVO) study. Circulation. 2012 Jun 19;125(24):2985-92.

85. Laoutaris ID, Adamopoulos S, Manginas A, Panagiotakos DB, Kallistratos MS, Doulaptsis C, et al. Benefits of combined aerobic/resistance/inspiratory training in patients with chronic heart failure. A complete exercise model? A prospective randomised study. International journal of cardiology. 2012 May 31.

86. Liu MH, Wang CH, Huang YY, Tung TH, Lee CM, Yang NI, et al. Edema index-guided disease management improves 6-month outcomes of patients with acute heart failure. International heart journal. 2012;53(1):11-7.

87. Lynga P, Persson H, Hagg-Martinell A, Hagglund E, Hagerman I, Langius-Eklof A, et al. Weight monitoring in patients with severe heart failure (WISH). A randomized controlled trial. European journal of heart failure. 2012 Apr;14(4):438-44.

88. Mann DL, Kubo SH, Sabbah HN, Starling RC, Jessup M, Oh JK, et al. Beneficial effects of the CorCap cardiac support device: five-year results from the Acorn Trial. The Journal of thoracic and cardiovascular surgery. 2012 May;143(5):1036-42.

89. Martin DO, Day JD, Lai PY, Murphy AL, Nayak HM, Villareal RP, et al. Atrial Support Pacing in Heart Failure: Results from the Multicenter PEGASUS CRT Trial. Journal of cardiovascular electrophysiology. 2012 Jun 27.

90. Masuyama T, Tsujino T, Origasa H, Yamamoto K, Akasaka T, Hirano Y, et al. Superiority of long-acting to short-acting loop diuretics in the treatment of congestive heart failure. Circulation journal : official journal of the Japanese Circulation Society. 2012;76(4):833-42.

91. Mello PR, Guerra GM, Borile S, Rondon MU, Alves MJ, Negrao CE, et al. Inspiratory muscle training reduces sympathetic nervous activity and improves inspiratory muscle weakness and quality of life in patients with chronic heart failure: a clinical trial. Journal of cardiopulmonary rehabilitation and prevention. 2012 Sep-Oct;32(5):255-61.

92. Park SJ, Milano CA, Tatooles AJ, Rogers JG, Adamson RM, Steidley DE, et al. Outcomes in advanced heart failure patients with left ventricular assist devices for destination therapy. Circulation Heart failure. 2012 Mar 1;5(2):241-8.

93. Parrinello G, Di Pasquale P, Torres D, Cardillo M, Schimmenti C, Lupo U, et al. Troponin I release after intravenous treatment with high furosemide doses plus hypertonic saline solution in decompensated heart failure trial (Tra-HSS-Fur). American heart journal. 2012 Sep;164(3):351-7.

94. Rodriguez-Gazquez Mde L, Arredondo-Holguin E, Herrera-Cortes R. Effectiveness of an educational program in nursing in the self-care of patients with heart failure: randomized controlled trial. Revista latino-americana de enfermagem. 2012 Mar-Apr;20(2):296-306.

95. Rogers DP, Lambiase PD, Lowe MD, Chow AW. A randomized double-blind crossover trial of triventricular versus biventricular pacing in heart failure. European journal of heart failure. 2012 May;14(5):495-505.

96. Sandri M, Kozarez I, Adams V, Mangner N, Hollriegel R, Erbs S, et al. Age-related effects of exercise training on diastolic function in heart failure with reduced ejection fraction: the Leipzig Exercise Intervention in Chronic Heart Failure and Aging (LEICA) Diastolic Dysfunction Study. European heart journal. 2012 Jul;33(14):1758-68.

97. Schou M, Gustafsson F, Videbaek L, Tuxen C, Keller N, Handberg J, et al. Extended heart failure clinic follow-up in low-risk patients: a randomized clinical trial (NorthStar). European heart journal. 2012 Aug 8.

98. Servantes DM, Pelcerman A, Salvetti XM, Salles AF, de Albuquerque PF, de Salles FC, et al. Effects of home-based exercise training for patients with chronic heart failure and sleep apnoea: a randomized comparison of two different programmes. Clinical rehabilitation. 2012 Jan;26(1):45-57.

99. Seto E, Leonard KJ, Cafazzo JA, Barnsley J, Masino C, Ross HJ. Mobile phone-based telemonitoring for heart failure management: a randomized controlled trial. Journal of medical Internet research. 2012;14(1):e31.

100. Smart NA, Haluska B, Jeffriess L, Leung D. Exercise Training in Heart Failure With Preserved Systolic Function: A Randomized Controlled Trial of the Effects on Cardiac Function and Functional Capacity. Congestive heart failure (Greenwich, Conn). 2012 Apr 26.

101. Soska V, Dobsak P, Pohanka M, Spinarova L, Vitovec J, Krejci J, et al. Exercise training combined with electromyostimulation in the rehabilitation of patients with chronic heart failure: A randomized trial. Biomedical papers of the Medical Faculty of the University Palacky, Olomouc, Czechoslovakia. 2012 Nov 1.

102. Stewart S, Carrington MJ, Marwick TH, Davidson PM, Macdonald P, Horowitz JD, et al. Impact of Home Versus Clinic-Based Management of Chronic Heart Failure: The WHICH? (Which Heart Failure Intervention Is Most Cost-Effective & Consumer Friendly in Reducing Hospital Care) Multicenter, Randomized Trial. Journal of the American College of Cardiology. 2012 Oct 2;60(14):1239-48.

103. Teerlink JR, Cotter G, Davison BA, Felker GM, Filippatos G, Greenberg BH, et al. Serelaxin, recombinant human relaxin-2, for treatment of acute heart failure (RELAX-AHF): a randomised, placebo-controlled trial. Lancet. 2012 Nov 6.

104. Veroff DR, Sullivan LA, Shoptaw EJ, Venator B, Ochoa-Arvelo T, Baxter JR, et al. Improving self-care for heart failure for seniors: the impact of video and written education and decision aids. Population health management. 2012 Feb;15(1):37-45.

105. Witham MD, Fulton RL, Greig CA, Johnston DW, Lang CC, van der Pol M, et al. Efficacy and cost of an exercise program for functionally impaired older patients with heart failure: a randomized controlled trial. Circulation Heart failure. 2012 Mar 1;5(2):209-16.

106. Wu JR, Corley DJ, Lennie TA, Moser DK. Effect of a medication-taking behavior feedback theory-based intervention on outcomes in patients with heart failure. Journal of cardiac failure. 2012 Jan;18(1):1-9.
